# S100 and p65 expression are increased in the masseter muscle after botulinum toxin-A injection

**DOI:** 10.1186/s40902-016-0079-8

**Published:** 2016-08-26

**Authors:** Young-Wook Park, Seong-Gon Kim, You-Young Jo

**Affiliations:** 1 Department of Oral and Maxillofacial Surgery, College of Dentistry, Gangneung-Wonju National University, 7 Jukhyun-gil, Gangneung, 210-702 South Korea; 2 Sericultural and Apicultural Materials Division, National Academy of Agricultural Science, Suwon, South Korea

**Keywords:** Botulinum toxin-A, p65, S100, Apoptosis, Masseter muscle

## Abstract

**Background:**

The purpose of this study was to compare the expression levels of p65 and S100 in the rat masseter muscle after the injection of different concentrations of botulinum toxin-A (BTX-A).

**Methods:**

We injected either 5 or 10 U of BTX-A into both masseter muscle of rats. As a control group, the same volume of saline was injected. After 14 days, the animals were sacrificed. Subsequently, a biopsy and immunohistochemical staining of the samples were performed using a p65 or S100 antibody.

**Results:**

The cross-sectional area of each myofibril was significantly reduced by BTX-A injection (*P* < 0.001). The expression of p65 and S100 increased significantly with increasing concentrations of BTX-A (*P* < 0.001).

**Conclusions:**

The injection of BTX-A into the masseter muscle induced muscle atrophy. Subsequently, p65 and S100 expression in myoblasts were increased for the protection of muscle cells.

## Background

Botulinum toxin is a neurotoxin produced by the anaerobic bacterium *Clostridium botulinum* [1, 2]. This toxin selectively hydrolyzes the receptor that is required for the binding of the synapse vesicle and the membrane on the pre-synapse of the neuromuscular junction; as a result, the toxin blocks the release of acetylcholine [3–6], causing muscle weakness, paralysis, and atrophy [7]. Botulinum toxin-A (BTX-A) is a type of botulinum toxin that was approved by the US Food and Drug Administration (FDA) in 1989. Since that time, BTX-A has been used for cosmetology, focal dystonia, facial spasm, hyperhidrosis, and the treatment of muscle hypertrophic disorder [8, 9].

In addition, in the oral and maxillofacial area, BTX-A is injected into masticatory muscles such as the masseter muscle or the temporal muscle for esthetic and therapeutic purposes [10–12]. Previous studies primarily investigated the effect of BTX-A on muscles and nerves [13]. However, muscle paralysis or weakness impacts on the bone, resulting in a reduction of the affected bone, even if BTX-A is injected into the muscle [14, 15]. Therefore, more studies are needed to explain the mechanism by which BTX-A affects the masseter muscle.

BTX-A injection into the masseter muscle induces muscle atrophy [16]. Several marker proteins have the potential to increase their expression during the skeletal muscle atrophy process [17]. S100 is a well-known calcium-binding protein [18, 19]. The terminal deoxynucleotidyl transferase dUTP nick end labeling (TUNEL) assay has been used to assess apoptosis in many publications [20]. After burn injury, muscle atrophy is accompanied by increasing S100 levels and TUNEL-positive cells in the motor nerve [21]. The NF-kB pathway is important in cellular apoptosis. The expression of caspases and key components of the NF-kB pathway (p65) is increased during skeletal muscle atrophy [22]. Therefore, the expression levels of S100 and p65 in the masseter muscle may change after BTX-A injection. However, S100 expression levels have not been studied before in the context of BTX-A injection.

The purpose of this study was to compare the expression levels of p65 and S100 in the rat masseter muscle after the injection of different concentrations of BTX-A.

## Methods

### Experimental animals and housing conditions

Fifteen Sprague-Dawley rats (age 10–12 weeks, body weights 250–300 g) were purchased from Samtako (Osan, Korea). Each rat was individually housed and allowed an adaptation period for 10 days. The Institutional Animal Care and Use Committee of Gangneung-Wonju National University approved this experiment (GWNU 2015-25).

### Experimental design

The animals were divided into three groups: the control group and two experimental groups. Five rats were used in each group. In one experimental group, 5 U of a BTX-A solution was injected into both sides of the masseter muscle. In the other experimental group, 10 U of a BTX-A solution was injected on both sides of the masseter muscle. The control group was injected with 0.1 cc of saline on both sides of the masseter muscle. Fourteen days after injection, all rats were sacrificed, and samples containing the masseter muscle and mandible were obtained for histological examination. After the harvested tissues were fixed in a formalin solution for 1 day, decalcification was performed with a 5 % nitric acid solution, and specimens containing the masseter muscle and mandible were generated. For following evaluation, the specimens were cut as horizontal plane. The height for sectioning ramus was determined as the central cut between the zygomatic arch plane and the lower mandibular border plane.

The specimens were stained with hematoxylin and eosin (H&E) to evaluate the cortical bone thickness of the mandibular ramus. We photographed the histological view at 14 days post-surgery and measured the ramal cortical bone thickness of the rats using size measuring software (SigmaScan-Pro®; SPSS Science, Chicago, IL, USA). The cross-sectional area of myofibrils was also measured using the same software. Immunohistochemical staining was performed for p65 and S100. Both antibodies were murine monoclonal antibodies purchased from Santa Cruz Biotech (Santa Cruz, CA, USA). The primary antibody dilutions were as follows: S100, 1:30; and p65, 1:50. Subsequent procedures were in accord with a previous publication [23].

To quantify the intensity of the immunohistochemical reaction, the intensity of positive staining was evaluated in five random fields of the masseter muscle at a ×200 magnification using a computer-assisted image analysis program. The staining intensity in immunohistochemistry experiments was shown as the mean intensity value (0: no stain, 255: highest stain). Counterstaining procedure was omitted to ensure that the intensity value would be solely attributable to the positive immunohistochemical reaction.

### Statistical analysis

All of the results were statistically analyzed using one-way analysis of variance (ANOVA), followed by a post hoc test (Bonferroni’s method). The significance level was set as *P* < 0.05.

## Results

**Fig. 1:**
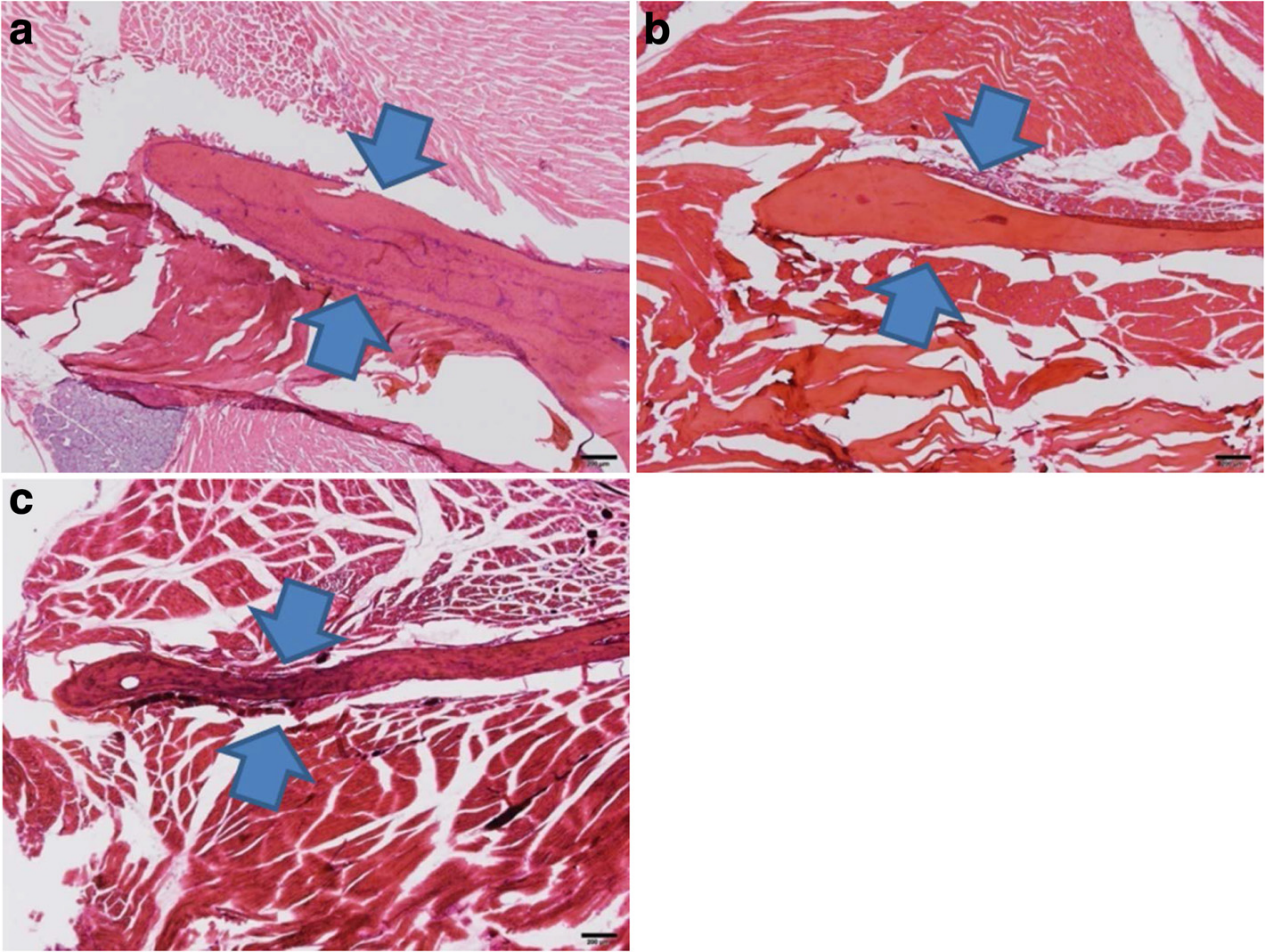
Cortical bone of the rat mandibular ramus. The *arrows* indicate the mandibular ramus. **a** Saline-treated group; **b** 5-U BTX-A-treated group; **c** 10-U BTX-A-treated group (H&E stain, original magnification ×20)

A horizontal cut of the mandibular ramus is shown in Fig. 1a–c for saline-treated, 5-U BTX-A-treated, and 10-U BTX-A-treated specimens, respectively. The average thicknesses of the cortical bone were measured as follows: the saline-treated control group had a thickness of 0.18 ± 0.05 mm, the 5-U BTX-A-treated group had a thickness of 0.15 ± 0.05 mm, and the 10-U BTX-A-treated group had a thickness of 0.10 ± 0.03 mm (Table 1). The cortical bone thickness of the mandibular ramus of rats was reduced in the BTX-A-treated groups in comparison to the saline-treated control group. However, the difference observed among the groups was not statistically significant (*P =* 0.055).

**Table 1:** The cortical bone thickness and the cross-sectional areas of masseter muscle after BTX-A injection

Group	Cortical bone thickness		Cross-sectional area	
	Average (mm)	*P* value	Average (μm^2^)	*P* value
Saline-treated	0.18 ± 0.05	–	621.68 ± 87.74	–
5-U BTX-A-treated	0.15 ± 0.05	NS	495.42 ± 110.15	0.037
10-U BTX-A-treated	0.10 ± 0.03	NS	263.05 ± 115.31	<0.001

The *P* value was calculated by post hoc test and compared with the saline-treated group

*BTX-A* botulinum toxin-A

**Fig. 2:**
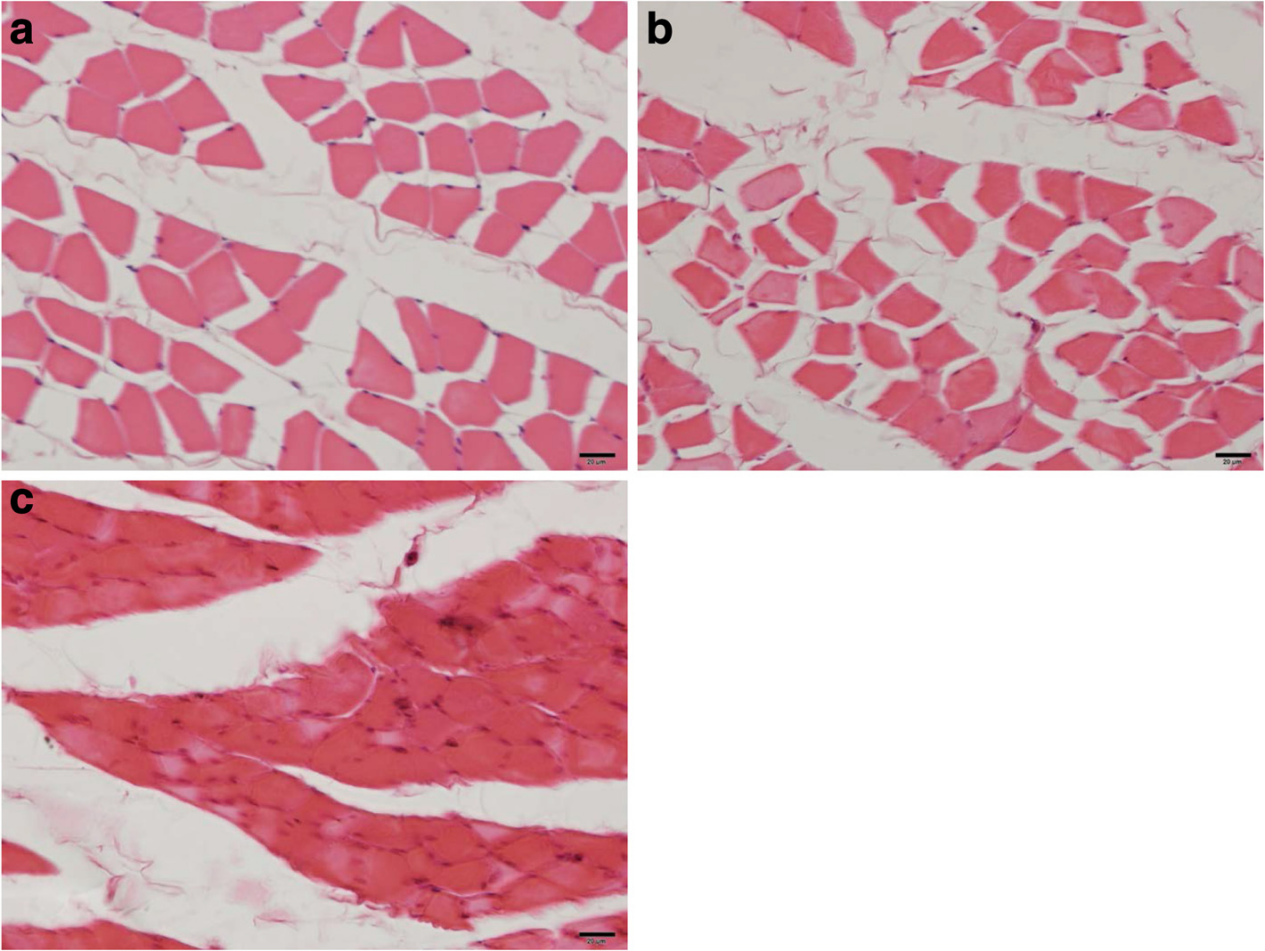
Cross section of the masseter muscle. **a** Saline-treated group; **b** 5-U BTX-A-treated group; **c** 10-U BTX-A-treated group (H&E stain, bar = 20 μm)

A cross section of the masseter muscle is shown in Fig. 2a–c for saline-treated, 5-U BTX-A-treated, and 10-U BTX-A-treated specimens, respectively. The average cross-sectional areas of myofibrils were 621.68 ± 87.74 μm^2^, 495.42 ± 110.15 μm^2^, and 263.05 ± 115.31 μm^2^ for the saline-treated control, the 5-U BTX-A-treated group, and the 10-U BTX-A-treated group, respectively (Table 1). The cross-sectional area of myofibrils was significantly reduced in the BTX-A-treated groups in comparison to the saline-treated control group (*P <* 0.001). The post hoc test revealed differences between the group treated with 10 U BTX-A and the other groups, with significantly lower values in the 10-U BTX-A group than in the saline-treated control and the 5-U BTX-A-treated group (*P <* 0.001 for both groups). When we compared the 5-U BTX-A-treated group to the saline-treated control, the observed difference was statistically significant (*P =* 0.037).

**Fig. 3:**
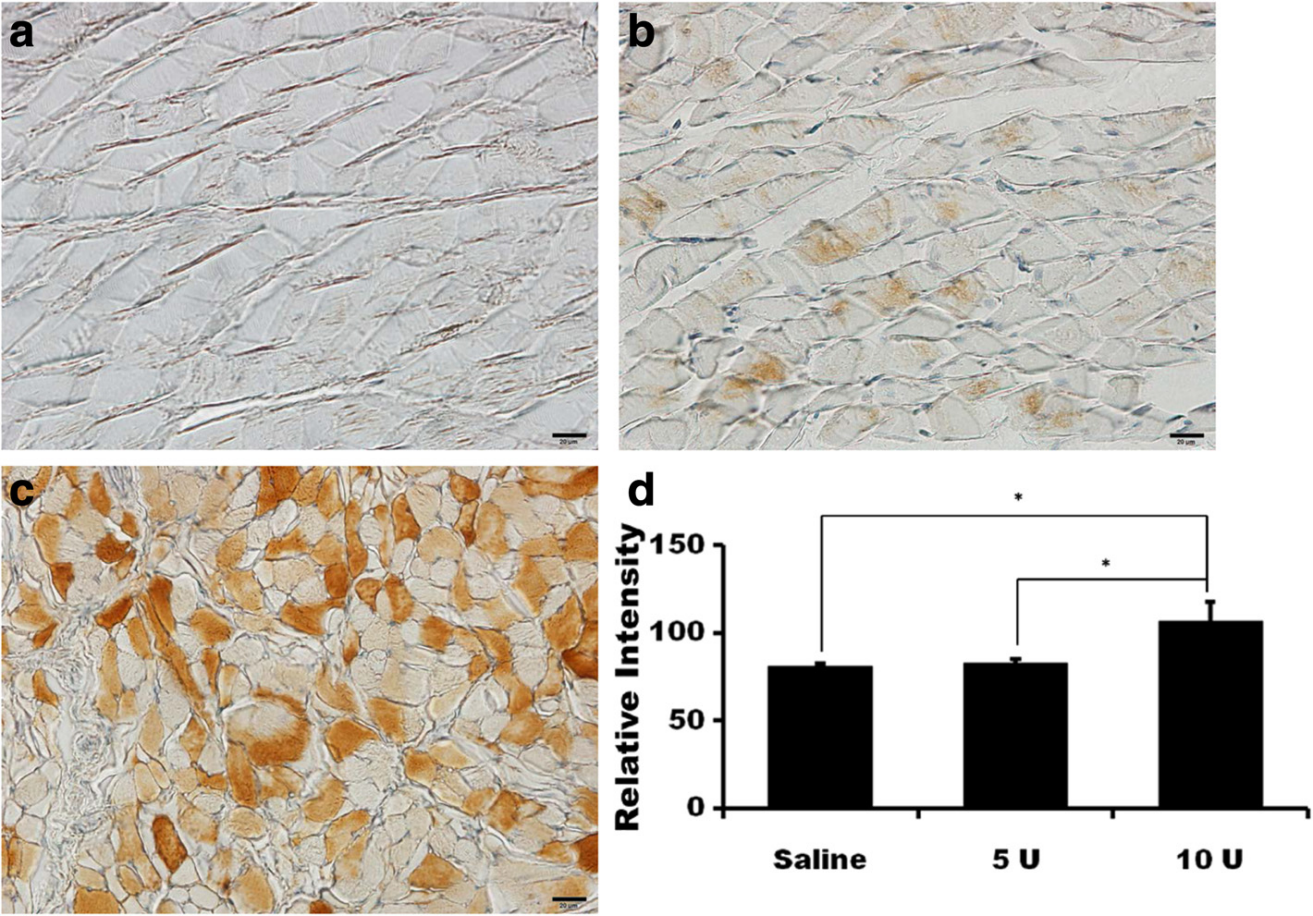
Immunohistochemical staining for p65. **a** Saline-treated group; **b** 5-U BTX-A-treated group; **c** 10-U BTX-A-treated group (without counterstaining, bar = 20 μm). **d** Measurement of the average intensity of staining (*P < 0.05)

**Fig. 4:**
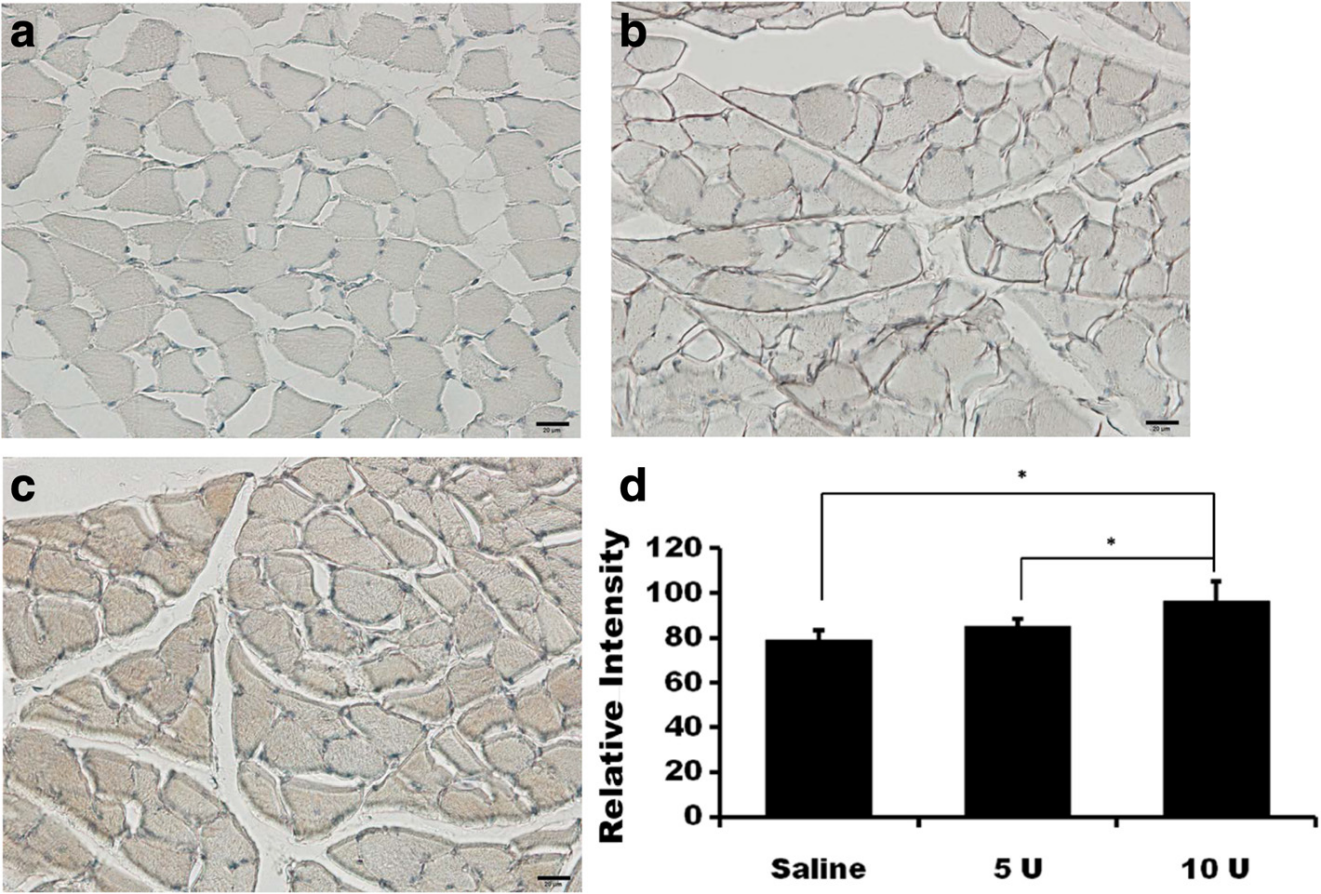
Immunohistochemical staining for S100. **a** Saline-treated group; **b** 5-U BTX-A-treated group; **c** 10-U BTX-A-treated group (without counterstaining, bar = 20 μm). **d** Measurement of the average intensity of staining (*P < 0.05)

The immunohistochemical findings for p65 and S100 are shown in Figs. 3 and 4. The immunohistochemical findings demonstrated that the expression of p65 and S100 were significantly higher in the 10-U BTX-A-treated groups than in the saline group (Figs. 3d and 4d; *P <* 0.001 and *P* = 0.002 for p65 and S100, respectively). The mean intensity values for p65 were 81.02 ± 1.53, 82.93 ± 2.11, and 106.46 ± 10.86 for the saline, 5-U BTX-A, and 10-U BTX-A treatments, respectively. The post hoc test revealed differences between the group treated with 10 U of BTX-A and the other groups, with significantly higher values in the 10-U BTX-A group than in the saline-treated control and the 5-U BTX-A-treated group (*P <* 0.001 for both groups). The mean intensity values for S100 were 78.98 ± 4.36, 85.31 ± 3.09, and 96.46 ± 8.98 for the saline, 5-U BTX, and 10-U BTX treatments, respectively. The post hoc test revealed differences between the group treated with 10 U BTX-A and the other groups, with significantly higher values in the 10-U BTX-A group than in the saline-treated control and the 5-U BTX-A-treated group (*P =* 0.002 and *P =* 0.038 for the saline- and 5-U BTX-A-treated groups, respectively).

## Discussion

BTX-A has been used in a variety of oral and maxillofacial applications. BTX-A is primarily injected into masticatory muscles, such as the masseter muscle or the temporal muscle, for esthetic and therapeutic purposes in the maxillofacial area [3, 6]. A small number of studies that investigated the effects of BTX on masticatory muscles have been reported [7, 17]. However, short-term molecular-level changes in the masseter muscle after BTX-A injection have not been reported previously. In this study, p65 and S100 were increased in a dose-dependent manner in the rat masseter muscle 14 days after the BTX-A injection (Figs. 3 and 4). As the muscle underwent atrophic changes (Fig. 2), the thickness of the ramal cortical bone decreased 14 days after the BTX-A injection (Fig. 1). To our knowledge, this is the first report of dose-dependent early change in the masseter muscle after BTX-A injection.

In this study, the thickness of the cortical bone in the mandibular ramus was decreased 14 days after BTX-A injection in comparison to the saline-treated control (Fig. 1). However, the difference observed among the groups was not statistically significant (Fig. 1d, *P >* 0.05). Previous publication demonstrates significant difference in bone thickness at 4 and 8 weeks after BTX-A injection [24]. This lack of significance might be due to the small sample size and short follow-up period. The thinner cortex of the mandibular ramus in the BTX-injected group might be similar to the disuse atrophy that occur secondary to masseter muscle atrophy. A number of previous studies reported that if the bone does not receive continuous stimulation from muscles, the bone will atrophy [8, 14]. When the patient having osteoporosis receives BTX-A injection, the optimal dosage of BTX-A should be carefully monitored. BTX-A injection results in an immediate reduction of electromyographic signals in the masseter muscle [5]. In this study, BTX-A-injected myofilaments exhibited a reduced size in comparison to the saline-injected control (Fig. 2).

Generally, muscle atrophy is induced by systemic illnesses that contribute to muscle wasting. In this study, BTX-A injection reduced the size of myofibrils; this change should be interpreted as muscle atrophy (Fig. 2). A component of NF-kB (p65) is activated during cytokine-induced myotubule atrophy [25]. In addition, serum S100 induces myoblast apoptosis via the stimulation of reactive oxygen species [26]. In this study, the expression of NF-kB and the S100 was increased in the BTX-A injected groups in comparison to the saline-treated control. When the apoptosis of cells occurs, the expression of NF-kB is increased [14, 27]. When the calcium concentration in the tissue is increased, the expression of S100 also is increased [28, 29]. The observed increase in both p65 and S100 might be caused by muscle atrophy or apoptotic stress after BTX-A injection. When muscle apoptosis occurs, intracellular calcium ions may be released into the extracellular space [30]. As S100 is a calcium-binding protein, increased calcium release may increase the expression of S100. However, S100 protects against myoblast apoptosis [31]. Thus, increased expression of S100 might occur due to the protection of myoblasts from apoptosis after BTX-A injection. BTX-A induced myoblast apoptosis is also confirmed in recent publication [32].

## Conclusions

In this study, the injection of BTX-A into the masseter muscle induced muscle atrophy. Subsequently, p65 and S100 expression in myoblasts were increased for the protection of muscle cells.
